# *US6* Gene Deletion in Herpes Simplex Virus Type 2 Enhances Dendritic Cell Function and T Cell Activation

**DOI:** 10.3389/fimmu.2017.01523

**Published:** 2017-11-10

**Authors:** Angello Retamal-Díaz, Kayla A. Weiss, Eduardo I. Tognarelli, Mariela Freire, Susan M. Bueno, Betsy C. Herold, William R. Jacobs, Pablo A. González

**Affiliations:** ^1^Millennium Institute on Immunology and Immunotherapy, Departamento de Genética Molecular y Microbiología, Facultad de Ciencias Biológicas, Pontificia Universidad Católica de Chile, Santiago, Chile; ^2^Department of Microbiology and Immunology, Albert Einstein College of Medicine, New York, NY, United States; ^3^Department of Pediatrics, Albert Einstein College of Medicine, New York, NY, United States; ^4^Department of Genetics, Albert Einstein College of Medicine, New York, NY, United States; ^5^Howard Hughes Medical Institute, Albert Einstein College of Medicine, New York, NY, United States

**Keywords:** dendritic cells, HSV type 2, glycoprotein D, apoptosis, adaptive immunity, unfolded protein response, migration

## Abstract

Herpes simplex virus (HSV) type 1 (HSV-1) and type 2 (HSV-2) produce lifelong infections that are associated with frequent asymptomatic or clinically apparent reactivation. Importantly, HSV express multiple virulence factors that negatively modulate innate and adaptive immune components. Notably, HSV interfere with dendritic cell (DC) viability and function, likely hindering the capacity of the host to mount effective immunity against these viruses. Recently, an HSV-2 virus that was deleted in glycoprotein D was engineered (designated ΔgD-2). The virus is propagated on a complementing cell line that expresses HSV-1 gD, which permits a single round of viral replication. ΔgD-2 is safe, immunogenic, and provided complete protection against vaginal or skin challenges with HSV-1 and HSV-2 in murine models. Here, we sought to assess the interaction of ΔgD-2 with DCs and found that, in contrast to wild-type (WT) virus which induces DC apoptosis, ΔgD-2 promoted their migration and capacity to activate naïve CD8^+^ and CD4^+^ T cells *in vitro* and *in vivo*. Furthermore, DCs exposed to the WT and ΔgD-2 virus experienced different unfolded protein responses. Mice primed with DCs infected with ΔgD-2 *in vitro* displayed significantly reduced infection and pathology after genital challenge with virulent HSV-2 compared to non-primed mice, suggesting that DCs play a role in the immune response to the vaccine strain.

## Introduction

Infections with herpes simplex viruses (HSV) are common, with two-thirds of the world population infected with HSV type 1 (HSV-1) and nearly 20% infected with HSV type 2 (HSV-2) (1–3). Infections are lifelong and virus shedding occurs continuously, both in symptomatic and asymptomatic individuals (4, 5). Because HSV infections may severely affect healthy and immunocompromised individuals and HSV-2 contributes significantly to HIV epidemics, novel vaccines against these viruses are urgently needed (4).

Recently, an HSV-2 virus that has the *U_S_6* gene deleted (encoding glycoprotein D, gD) phenotypically complemented by growing the virus on a cell line that expresses HSV-1 gD (VD60 cells) was reported to be safe and immunogenic in mice. The vaccine strain (designated ΔgD-2) conferred complete protection against skin and vaginal challenges with high doses of clinical isolates of HSV-1 and HSV-2 and prevented the establishment of latency (6, 7). An important characteristic of ΔgD-2 is that it is restricted to a single cycle, because progeny do not express gD which is required for viral entry and cell-to-cell spread (8).

Dendritic cells (DCs) are key immune cells with the capacity to sense, process, and present microbe-derived components to T cells for eliciting cytotoxic CD8^+^ T lymphocytes and helper T cells (CD4^+^), which can support the production of high-affinity antiviral antibodies and exert direct antiviral functions (9). Notably, HSV-1 and HSV-2 infect DCs *in vitro* and negatively modulate their maturation, migration, and autophagosome activity and promote their apoptosis (10–14). These responses may contribute to immune evasion and decrease the host’s capacity to establish an effective antiviral response (11, 15–20).

We hypothesize that ΔgD-2 may differentially affect DC function as compared to wild-type virus (WT) or other single cycle vaccine candidate HSV variants and that these differences may contribute to the immunogenicity of the vaccine. To test this hypothesis, we compared the response of murine DCs to ΔgD-2, a single cycle strain deleted in glycoprotein H (herein ΔgH-2) (21), viruses deleted in other non-essential glycoproteins (glycoprotein I and glycoprotein J) and WT virus. When cocultured with T cells, DCs infected with ΔgD-2 activated virus-specific CD8^+^ T cells and antigen-specific CD4^+^ T cells both, *in vitro* and *in vivo*. Animals inoculated with ΔgD-2 in the footpads displayed increased numbers of DCs migrating from the injection site to lymph nodes (LNs) as compared to animals inoculated with the WT virus. Furthermore, animals that received DCs that were inoculated *in vitro* with ΔgD-2 manifested reduced pathology and decreased viral loads in vaginal lavages (VALs) and the dorsal root ganglia (DRG) after genital challenge with a lethal dose of HSV-2. In contrast, the ΔgH-2 virus behaved similarly to the WT virus in DCs, and when mice were treated with DCs infected with ΔgH-2 *in vitro*, a more modest antibody response was observed and the DC immunization conferred only partial protection to the genital challenge with HSV-2. Taken together, these results suggest that ΔgD-2 activates DCs to promote antigen presentation and that this may contribute to the unique immunogenicity and protective capacity of this candidate vaccine strain.

## Materials and Methods

### Mice

Mice were harbored in pathogen-free facilities at the Pontificia Universidad Católica de Chile (Santiago, Chile). Female C57BL/6J mice were obtained from the Jackson Laboratory (Bar Harbor, ME, USA). The gBT-I transgenic mouse strain, encoding an HSV-specific T cell receptor that recognizes H-2K^b^(MHC-I)/gB_(498-505)_, was kindly shared by Dr. Francis Carbone (22) and provided by Dr. Akiko Iwasaki at Yale University, New Haven, CT, USA. The OT-II transgenic mouse strain, encoding a T cell receptor that recognizes I-A^b^(MHC-I)/OVA_323–339_, was originally obtained from Dr. Ralph Steinman at the Rockefeller University, New York, NY, USA, and kindly shared with us by Dr. Alexis Kalergis, P. Universidad Católica de Chile. B6.SJL-*Ptprc^a^Pepc^b^*/BoyJ mice (CD45.1^+^) were obtained from the Jackson Laboratory. Mice were handled and euthanized according to the guidelines of the Institutional Ethics Committee and according to the approved protocol CBB-201/2013.

### Virus Preparation

VD60 cells were used to propagate the HSV-2 ΔgD-2 virus as previously described (18, 23). Vero cells (ATCC CCL-81) were used to propagate and titer the HSV-2(G) (parental strain of the ΔgD-2 virus), as well as the ΔgI-2 and ΔgJ-2 viruses and their parental strain HSV-2-BAC38 (H312-1) (24). The ΔgI-2 and ΔgJ-2 viruses were generated using the methodology described by Cheshenko et al. with the primers described in Table S1 in Supplementary Material to create the allelic exchange substrate (25). Loss of the *sacB*-hygromycin cassette was verified by PCR with primers LL plus RR (Table S1 in Supplementary Material) for each gene replacement. Primers LL-Van91I-US5 and RR-Van91I-US5 (Table S1 in Supplementary Material) were used to check resolution of the *sacB*-hygromycin cassette in p0004S-Δ*US5*. Primers LL-Van91I-US7 and RR-Van91I-US7 were used to check resolution of the *sacB*-hygromycin cassette in p0004S-Δ*US7*. One microgram of the mutated HSV-2 BAC DNA, which contains a GFP reporter gene was transfected into Vero cells with Effectene (Qiagen). Two to three days after transfection, plates were screened for green fluorescent virus plaque formation and supernatants from positive wells were overlaid over fresh Vero cells for 1 h. Cells were then washed and covered with 1% low-melting agarose prepared in Opti-MEM (Thermo Fisher Scientific). Single green fluorescent plaques were picked and purified three times using this method. The previously described ΔgH-2 was propagated on Vero cells encoding HSV-1 gH (F6 cells) (26) as previously described (25). HSV-2 WT^+gD-1^ corresponds to WT HSV-2 virus propagated on VD60 cells and thus this virus will express both gD-2 and gD-1 on the virion surface. ΔgD-2^−/+gD-2^ corresponds to the ΔgD-2 virus propagated on ET60 cells. ET60 cells are Vero cells transduced with a lentivirus that encodes gD-2 from the HSV-2 G strain, which was cloned at the *XbaI* and *BamHI* sites of the pULTRA plasmid (pUltra was a gift from Malcolm Moore, Addgene plasmid # 24129) (27). Titration of the abovementioned viruses was performed over Vero cells (for WT, ΔgI-2, and ΔgJ-2) or glycoprotein-complementing cells (WT^+gD-1^, ΔgD-2, ΔgD-2^−/+gD-2^, and ΔgH-2). Uninfected Vero and VD60 cells were used as controls in experiments (mock infections).

### DC Infection with HSV and Viability

Dendritic cells were differentiated from bone marrow precursors of C57BL/6 mice as previously described (28). After 5 days of culture, DCs were inoculated for 1 h at 37°C with WT, WT^+gD-1^, ΔgD-2, ΔgD-2^−/+gD-2^, ΔgH-2, ΔgI-2, or ΔgJ-2 at a multiplicity of infection (MOI) of 1 or 10, as indicated. At 6, 12, 24, and 48 h post-inoculation (hpi), DCs were collected and analyzed for infection and viability. DC viability was assessed by flow cytometry with the Zombie-NIR Fixable Viability Kit (BioLegend) and anti-CD11c and anti-I-A^b^ antibodies (BioLegend) after fixation with 2% paraformaldehyde (PFA) in a FACS CANTO II flow cytometer (BD Biosciences, USA). DC infection with HSV was assessed by extracting total DNA from infected cells and conducting a qPCR using 200 ng of DNA per reaction with a probe for the *UL30* gene. The following primers and probe were used: Fwd-GGCCAGGCGCTTGTTGGTGTA, Rev-ATCACCGACCCGGAGAGGGA, and Probe-CCGCCGAACTGAGCAGACACCCGC using an Applied Biosystems StepOnePlus thermocycler, as previously described (7, 29). Viral protein expression was analyzed by western blot using primary antibodies against anti-ICP5 (Santa Cruz), anti-VP16 (Santa Cruz), anti-ICP27 (Santa Cruz), and anti-β-actin (BioLegend). Caspase activity was pharmacologically inhibited by pretreating DCs for 1 h with 100 µM of the Pan caspase inhibitor Z-VAD-FMK (Tocris, Cat. #2163) or 50 µM of the Caspase-1 inhibitor Ac-YVAD-CMK (Cayman Chemicals, CAS #178603-78-6) (30). After adding these drugs, DCs were inoculated with viruses as described above.

### DC Maturation

Dendritic cell maturation was assessed by evaluating the surface expression of H-2K^b^ (MHC-I), I-A^b^ (MHC-II), CD80, and CD86 over CD11c^+^ cells by flow cytometry. All antibodies were purchased from BioLegend. IL-6, TNF-α, and IL-12 cytokine release was determined by ELISA 24 h after DC treatment. Recombinant murine IL-6, IL-10, TGF-α, and IL-12 (PeproTech) were used as standards for cytokine quantification. Production of reactive oxygen species (ROS) by DCs was measured using CellROX deep red reagent (Life Technologies) according to the manufacturer’s instructions and analyzed using a Cytation 5 Cell Imaging Multi-Mode Reader (BioTek).

### DC-DC Cocultures

Dendritic cells were differentiated from bone marrow precursors of C57BL/6 (B6, CD45.1^−^) and B6 CD45.1 (CD45.1^+^) mice, as described above. At day 5, CD45.1^−^ DC preparations were inoculated with WT, ΔgD, or ΔgH-2 at MOI equal to 10 for 1 h at 37°C. After 24 h, CD45.1^+^-DC were added to the culture of virus-inoculated CD45.1^−^ DCs and then incubated for an additional 24 h at 37°C. DC viability was analyzed by flow cytometry with the Zombie-NIR Fixable Viability Kit (BioLegend), anti-CD11c, anti-I-A^b^, anti-CD45.1 anti-H-2K^b^, CD80, CD83, and CD86 (all from BioLegend); DCs were washed with phosphate-buffered saline (PBS), fixed with 2% PFA in PBS, and analyzed by flow cytometry in a Fortessa flow cytometer (BD Biosciences, USA).

### Electron Microscopy

Virus-inoculated DCs were prepared for transmission electron microscopy (TEM) at 12 h post-infection with 2.5% glutaraldehyde in sodium cacodylate (0.1 M), followed by treatment with 1% OsO_4_ and dehydration in acetone. Samples were then embedded in Epon-acetone resin and polymerized at 60°C for 24 h. Thin sections of 85 nm were prepared on an ultramicrotome and stained with 4% uranyl acetate in ethanol and lead citrate as previously described by others (31). Samples were observed using a Philips Tecnai 12 BioTWIN transmission electron microscope at 80 kV. For analyses of cell supernatants, DCs were inoculated for 1 h at 37°C with WT or ΔgD-2 at an MOI of 1. To determine if the visualized viral structures corresponded to *de novo* viral particle synthesis, DCs were inoculated with ΔgD-2 and WT virus in the presence of acyclovir 1 mg/ml (Pfizer). At 18 h post-infection, supernatants were collected and centrifuged for 10 min at 300 *g*, filtrated through a 0.45-µm filter and then ultracentrifuged at 119,000 *g* in a SW40 Ti rotor using an XPN 100K Beckman Coulter Ultracentrifuge. Supernatant concentrates were resuspended in 50 µl of PBS with protease inhibitors and characterized by negative staining using uranyl acetate for 1 min in the transmission electron microscope indicated above. Quantification of viral particles and viral genome copies in the viral structures recovered from the ultracentrifugated supernatants of DCs was performed by western blot using the antibody described above against ICP5 and by qPCR with the abovementioned primers and probe.

### Unfolded Protein Response (UPR)

Unfolded protein response in DCs was measured by qPCR on RNA extracted from DCs using TRIzol reagent (Thermo Fisher Scientific). Extracted RNA was reverse-transcribed using M-MLV and random primers (Promega, USA). TaqMan primers and probes were acquired from Applied Biosystems (Thermo Fisher Scientific) to evaluate BiP (Mm00517691_m1), Hsp90 (Mm00441926_m1), EDEM-1 (Mm00551797_m1), and CHOP (Mm01135937_g1) expression. GADPH was used as a housekeeping gene for normalization (Mm03928990_g1). To analyze XBP-1 splicing, we used primers that detect spliced and unspliced XBP-1 transcripts by qPCR, as previously described (32). Activation of the UPR PERK pathway was analyzed by western blot using anti-Phospho-eIF2ɑ and anti-eIF2ɑ antibodies (both from Cell Signaling).

### DC-T Cell Antigen Presentation Assays

Dendritic cells were inoculated at a MOI of 1 with the different viruses and collected 6 h later and cocultured with 1 × 10^5^ T cells/well; the T cells were either gBT-I CD8^+^ T cells purified from spleens of gBT-I transgenic mice using a CD8^+^ T cell negative selection kit (MiltenyiBiotec), or OT-II CD4^+^ T cells purified from the spleens of OT-II transgenic mice using a similar CD4^+^ T cell negative selection kit. For DC-OT-II cocultures, 200 nM ovalbumin_323–339_ peptide (pOVA_323–339_, Genscript) was added to the wells. After 24 h (gBT-I) or 48 h (OT-II) of coculturing, T cell activation, and differentiation was determined by ELISA by measuring IL-2, IL-4, and IFN-γ in the supernatants (28), and by flow cytometry using antibodies against CD4, CD8, CD25, and CD69 (all from BioLegend).

### DC Migration and T Cell Activation *In Vivo*

To evaluate the ability of DCs to migrate to draining LNs after viral inoculation, mice were inoculated subcutaneously in the hind limb or the footpad. For hind limb-inguinal LN DC follow-up, PBS (naïve), VD60 cellular lysates (mock), or 5 × 10^6^ PFU of either WT HSV-2 or ΔgD-2 was inoculated subcutaneously in 100 µl in the hind limb. Two days later, the inguinal LNs were removed and a single cell suspension was created by pressing the LNs between two sterile, frosted glass slides. Then, cells were counted, stained with anti-CD11c, CD80, CD86, I-A/I-E (MHC-II), CD40, CD90, F4/80, CD4, or CD8 antibodies in ice, and then washed and fixed with 1-step Fix/Lyse (eBioscience). Samples were analyzed in an LSR-II flow cytometer (BD Biosciences). For footpad-popliteal LN DC follow-up, mice were anesthetized and footpads were injected with 0.5 mM of 5- and 6-carboxyfluorescein diacetate succinimidyl ester (CFSE) (33) co-administered with 1 × 10^6^ PFU of WT or ΔgD-2 in 30 µl final volume. As a negative control, mice were injected with CFSE in PBS. As a positive control, mice were injected with 50 µg of lipopolysaccharide (LPS). At 24 h post-inoculation, mice were euthanized, and popliteal LNs were surgically removed to evaluate the presence of DCs with antibodies against CD11c and I-A^b^ by flow cytometry using a FACSCanto II flow cytometer (BD Biosciences). For assessing *in vivo* T cell activation, mice were inoculated in the hind limb as indicated above, and inguinal LNs were processed 2 days later for evaluating the following surface markers: CD4, CD8, CD69, and CD90. After staining as described above, cells were fixed with 1-step Fix/Lyse (eBioscience) and then analyzed in an LSR-II flow cytometer.

### Injection of DCs Inoculated *In Vitro* with Viruses and Genital Challenge with Virulent HSV-2

To assess whether DCs inoculated with ΔgD-2 *in vitro* could prime a protective immune response against a genital lethal challenge with HSV-2, DCs were isolated from the spleens of naïve C57BL/6 mice using a pan DCs isolation kit according to the manufacturer’s instructions (Miltenyi Biotec), and then inoculated *in vitro* with ΔgD-2, ΔgH-2, or UV-inactivated ΔgD-2 at an MOI 5 following the procedure described in Section “DC Infection with HSV and Viability.” Six hours later, DCs were transferred thrice into 6- to 8-week-old female C57BL/6 mice (1 × 10^6^ cells/mouse) subcutaneously on days 1, 7, and 14, respectively. Two weeks after the last injection of DCs, animals were challenged intravaginally with 5 × 10^5^ PFU of HSV-2 strain G (equivalent to 10× LD_90_), as previously described (6). To guarantee genital infection in all the mice, 5 days prior to the genital challenge animals were subcutaneously injected in the hind limb with medroxyprogesterone (2 mg/mouse). Sera were recovered 7 days after infection and compared with pre-immunization sera. Antibodies against HSV-2 were detected by ELISA, as described in Ref. (6) with minor modifications. Briefly, ELISA plates were coated with 0.75 µg of protein extracts, obtained either from HSV-2-infected Vero cells or non-infected cells, blocked with PBS-FBS 10% and then incubated with serial dilutions of the sera. To reduce non-specific antibody binding to the infected protein extracts, the sera were pre-adsorbed over uninfected Vero protein extracts for 2 h and then incubated with virus-infected cell extracts. After repetitive washes with PBS-Tween 20 0.05% (Thermo ELISA plate washer Wellwash Versa), the wells were incubated with an anti-mouse-IgG antibody conjugated to HRP (dilution 1:4,000), washed 7 times with PBS-Tween 0.05%, developed with TMB substrate (0.1 mg/ml) and read on a Multiskan ELISA plate reader at 450 nm after adding H_2_SO_4_ 2N to stop the enzymatic reaction. HSV-2 disease was followed in challenged mice for 11 days and animals were scored at the epithelial and neurological level as previously described (6). Viral loads in VALs were assessed at days 2, 4, and 11 by titering the recovered virus over monolayers of Vero cells. At day 11, dorsal root ganglia (DRG) were recovered and total DNA extracted with phenol-chloroform to determine viral genome loads by qPCR as described above in Section “DC Infection with HSV and Viability.”

### Statistical Analysis

Statistical significance between experimental groups was assessed either by unpaired Student’s *t*-test (bar graphs), one-way analysis of variance (ANOVA) with Bonferroni’s multiple comparison test (three or more groups), or two-way ANOVA with Tukey’s multiple comparison test (two independent variables, with a confidence interval of 95%) where indicated, using GraphPad Prism (GraphPad Software).

## Results

### HSV-2 ΔgD-2 Does Not Induce DC Death

**Figure 1:**
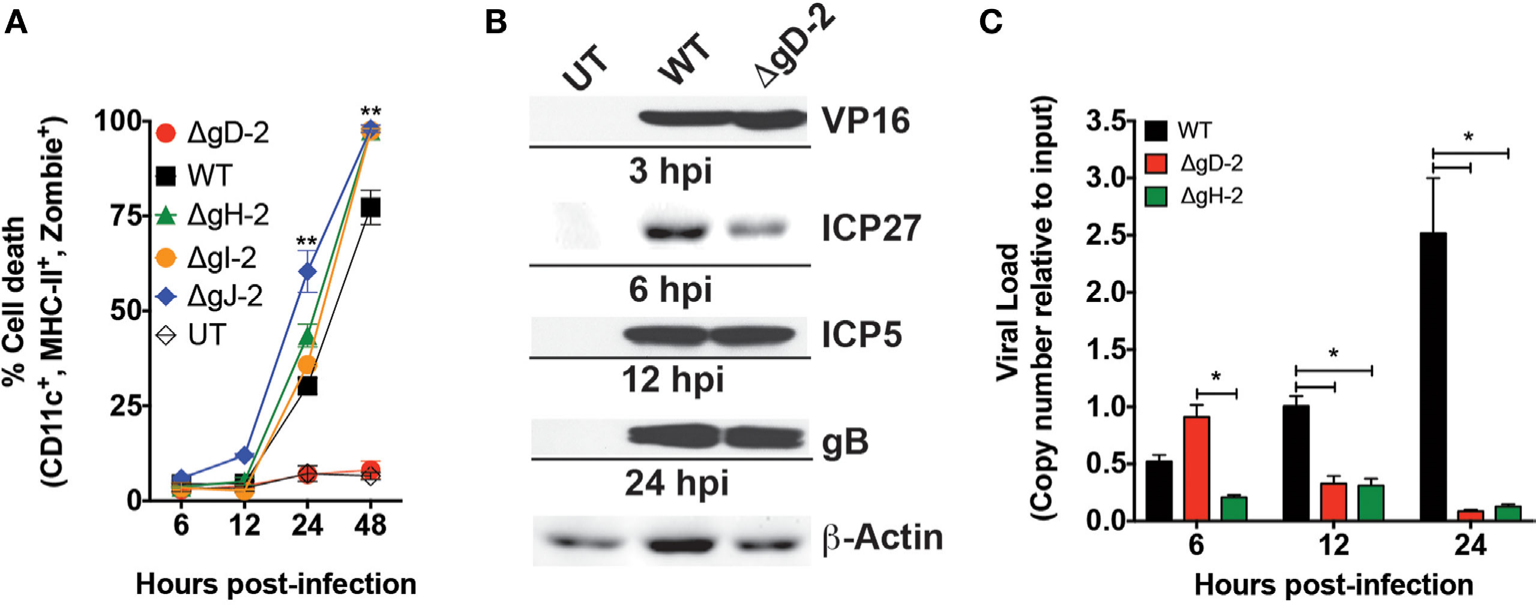
ΔgD-2 HSV type 2 (HSV-2) is attenuated in dendritic cells (DCs). **(A)** DC viability, as determined by flow cytometry (gated on CD11c^+^, I-A^b+^, Zombie^+^ cells) at different time points after virus inoculation with an multiplicity of infection (MOI) of 1. UT, untreated. **(B)** Western blot analyses of structural (VP16), early (ICP5, ICP27), and late (gB) viral proteins post-inoculation of DCs. **(C)** Viral genome loads in inoculated DCs determined by qPCR for the *UL30* gene. Data are means ± SEM of three independent experiments. Representative images are shown for western blots. Two-way analysis of variance and Tukey’s multiple comparison test were used for statistical analyses (**p < 0.01).

Previous studies indicate that HSV-2 elicits significant apoptosis in DCs (11–13, 17–19). To assess whether the ΔgD-2 mutant also alters the viability of these cells, murine DCs were exposed to WT, or ΔgD-2 virus and followed for 48 h, during which time cell viability was determined by flow cytometry. DCs infected with WT virus showed cell death at 24 hpi, which increased to nearly 80% by 48 h, consistent with previous reports (11, 17–19). A similar response was observed when DCs were infected with HSV-2 variants deleted in gH, gI, or gJ (ΔgH-2, ΔgI-2, and gJ, respectively). In contrast, ΔgD-2 elicited little or no cell death (Figure 1A). The contrasting response to ΔgD-2 versus ΔgH-2, which are both single cycle viruses, suggests that the phenotype of inducing DC death maps to gD. To determine whether reduced DC death by ΔgD-2 related to a loss of function of gD-2, because of the *U_s_6* gene deletion or to a gain of function due to gD-1 expression in this virus, we propagated the WT virus in VD60 cells (WT^+gD-1^) and the ΔgD-2 virus in a gD-2-complementing cell line (ET60 cells, ΔgD-2^−/+gD-2^ virus) and assessed their capacity to induce DC death. As shown in Figure S1A in Supplementary Material, the WT^+gD-1^ virus had the same effect as the WT virus over DC viability and the ΔgD-2^−/+gD-2^ virus behaved similarly to ΔgD-2. Taken together, these results suggest that attenuation of the ΔgD-2 virus in DCs is likely a consequence of the gD-2 deletion in this mutant virus.

To exclude the possibility that the increase in DC viability in response to ΔgD-2, compared to WT virus (or other deletion viruses) resulted from the detection of non-infected cells in the culture (e.g., bystander DCs) at an MOI that only infects a fraction of cells (MOI of 1), we performed a similar assay with an MOI of 10, which results in the infection of most DCs in the culture (11, 14). In this case, DCs inoculated with ΔgD-2 displayed significantly higher viability at 24 hpi as compared to DCs infected with the WT virus, which induced significant cell death (Figure S1B in Supplementary Material). Furthermore, we evaluated the survival of CD45.1^+^ DCs cocultured over WT-, ΔgH-, and ΔgD-2-virus inoculated CD45.1^−^ DCs 24 h after infection to determine the effect of DC infection on overlaid bystander DCs. We observed that DCs inoculated with the WT and ΔgH-2 elicited significant death of CD45.1^+^ DCs, indicating that their progeny viruses or soluble factors secreted by the corresponding DCs are toxic for accompanying cells (Figure S1C in Supplementary Material). However, this was not the case for DCs inoculated with the ΔgD-2 virus, which did not alter the viability of DCs cultured over previously virus-inoculated DCs. In addition, we tested the capacity of caspase inhibitors to restore the viability of DCs inoculated with WT or ΔgH-2 to assess their functions in subsequent assays. However, DCs inoculated with these viruses in the presence of these drugs succumbed to death, indicating that the mechanism by which HSV-2 kills DCs likely involves multiple pro-death signaling pathways (Figures S1D,E in Supplementary Material).

To compare the infectivity of ΔgD-2 and WT virus in DCs, we measured HSV-2 proteins (Figure 1B) and gene expression (Figure 1C) over a 24 h period, after infecting cells with MOI of 1. As shown in Figure 1B, the structural viral protein VP16, which is present in the virions was detectable at 3 hpi in cells inoculated with WT or ΔgD-2 viruses. Similarly, immediate early gene products (ICP5 and ICP27) and late (gB) gene products were detected in DCs infected with the WT and ΔgD-2 virus (Figure 1C). However, DCs infected with the WT virus contained increasing viral genome copy numbers over time which peaked at 24 h (Figure 1C), whereas ΔgD-2 and ΔgH-2 virus displayed significantly fewer genome copy numbers which dropped to undetectable levels by 24 h post-inoculation. These findings are consistent with the requirement for gD and gH for viral entry and cell–cell spread.

### Maturation of Virus-Inoculated DCs

**Figure 2:**
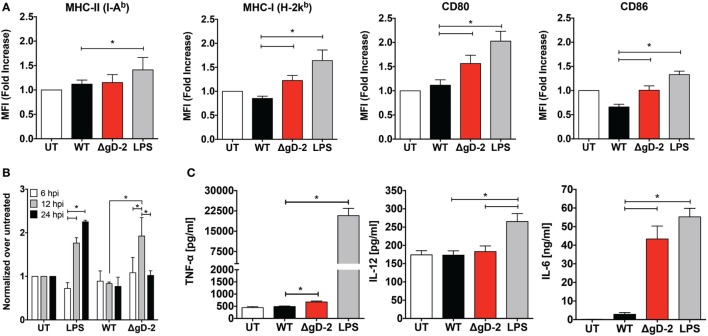
HSV type 2 (HSV-2) ΔgD-2 induces dendritic cell (DC) maturation and cytokine secretion. **(A)** Expression of surface maturation markers CD80, CD86, MHC-I (H-2K^b^), and MHC-II (I-A^b^), determined by flow cytometry on DCs (gated on CD11c^+^, Zombie^-^ cells) at 24 h post-virus inoculation. **(B)** Flow cytometry analysis of reactive oxygen species (ROS) production in DCs treated as indicated. **(C)** Supernatants from virus-inoculated DCs were assessed by ELISA for the presence TNF-α, IL-12, and IL-6. Data shown are means ± SEM from eight **(A)** and three **(B,C)** independent experiments. Two-way analysis of variance and Tukey’s multiple comparison test were used for statistical analyses (*p < 0.05).

To determine the impact of ΔgD-2 on DC maturation, we assessed MHC-I (H-2K^b^), MHC-II (I-A^b^), CD80, and CD86 expression by flow cytometry in DCs 24 h after viral inoculation. LPS, a toll-like receptor agonist that induces DC maturation, was included as a positive control. DCs infected with ΔgD-2 displayed a modest increase in some of the maturation markers assessed as compared to DCs infected with WT virus, specifically MHC-I, CD80, and CD86 (Figure 2A). Additionally, we evaluated these maturation markers and CD83 on CD45.1^+^ DCs cultured over WT-, ΔgH-, and ΔgD-2-infected CD45.1^−^ DCs 24 h after infection to determine the effect of infected DCs on bystander DCs (Figure S2 in Supplementary Material). Under this setting, only DCs infected with the WT virus displayed reduced MHC-I expression. Noteworthy, cytoplasmic ROS production in DCs, which can be used as a marker of DC maturation (34), was transiently increased at 12 h post-ΔgD-2 inoculation, which was not observed for DCs infected with the WT virus (Figure 2B). On the other hand, LPS, which was used as a positive control for DC maturation elicited sustained ROS production. Furthermore, we assessed the secretion of TNF-α, IL-12, and IL-6 by virally infected DCs. While a modest increase in TNF-α secretion was observed for DCs inoculated with ΔgD-2, the secretion of IL-12 was equivalent among all treatments (Figure 2C). Noteworthy, DCs exposed to ΔgD-2 produced significantly more IL-6 than DCs infected with WT virus. Again, to determine whether increased IL-6 secretion by DCs inoculated with ΔgD-2 was due to a loss of function because of its inability to express gD-2, or to a gain of function due to gD-1 expression, we assessed IL-6 secretion by DCs inoculated with the WT^+gD-1^ and ΔgD-2^−/+gD-2^ viruses. As shown in Figure S3 in Supplementary Material, DCs inoculated with the WT^+gD-1^ virus behaved similarly to cells infected with the WT virus and the DC response to ΔgD-2^−/+gD-2^ was equivalent to that of ΔgD-2, suggesting that in this case IL-6 secretion by DCs infected with ΔgD-2 is independent of the complementing glycoprotein required for initial entry of particles.

### DCs Inoculated with WT and ΔgD-2 HSV-2 Produce Defective Viral Particles

**Figure 3:**
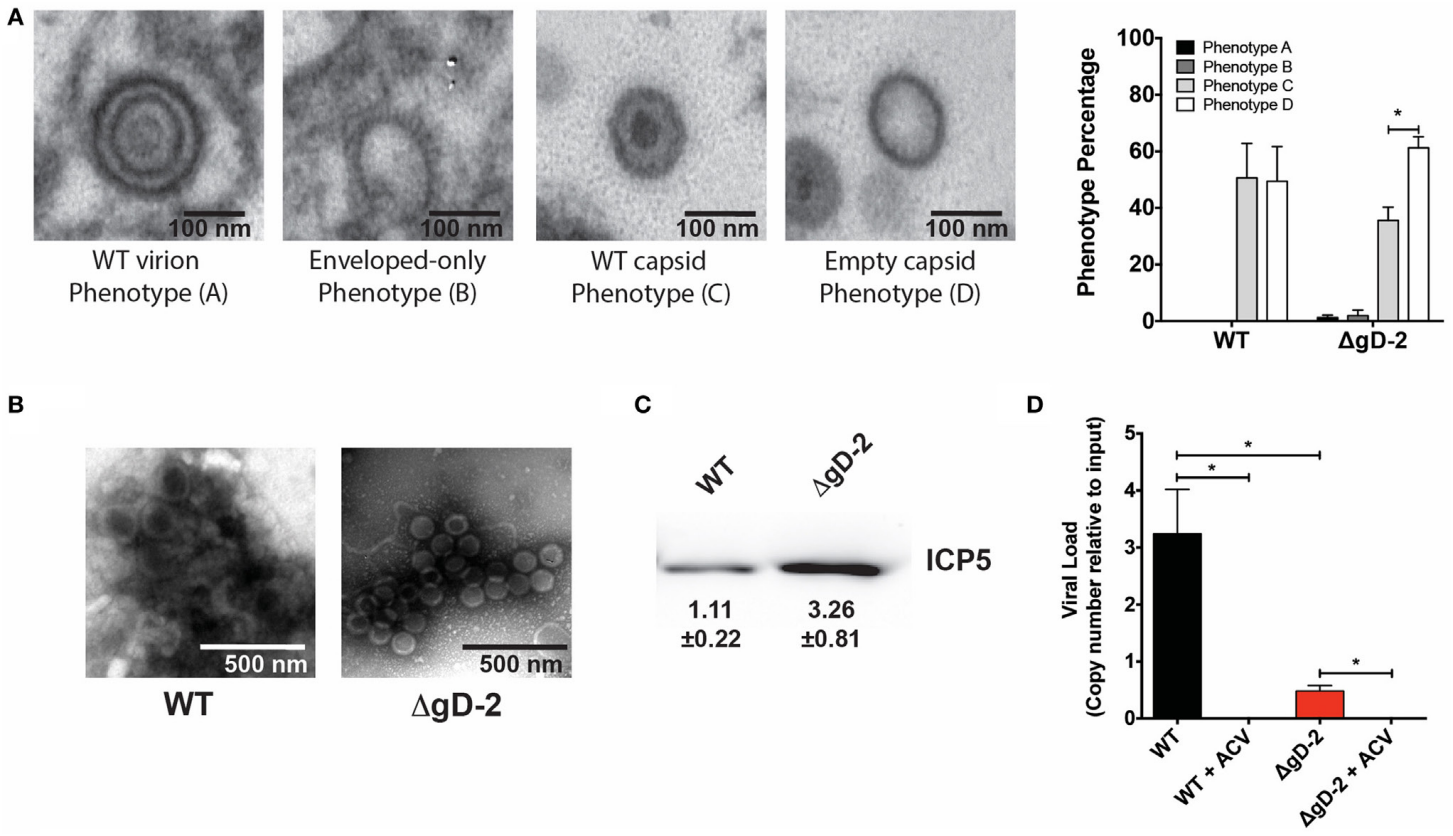
Dendritic cells (DCs) inoculated with ΔgD-2 or wild-type (WT) virus mainly release defective herpes simplex virus particles. **(A)** Four predominant viral particle phenotypes, indicated in the four panels, were observed in the extracellular space of virus-inoculated DCs (extracellular space data shown). Transmission electron microscopy. Extreme left: WT-like particles displaying an envelope, an electron-dense tegument, and an electron-dense capsid; middle left: envelope-only phenotype displaying an envelope-like structure, harboring spikes in the outer region, yet no apparent capsid or tegument; middle right: WT-like capsids, yet no envelope; extreme right: empty capsids with no envelope and no electron-dense content in capsids. Right panel: quantification of viral particle phenotypes observed. **(B)** Transmission electron microscopy (negative staining) of viral particles recovered from ultracentrifugated supernatant of virus-inoculated DCs. **(C)** Western blot analysis of the major capsid protein ICP5 in virus particles recovered from ultracentrifugated supernatants of DCs 18 h post-virus inoculation. Values indicate relative expression of ICP5. **(D)** Quantification of viral genome copies (determined by qPCR) in viral particles obtained from ultracentrifugated supernatants derived from virus-inoculated DCs with or without acyclovir treatment. One-way analysis of variance and Tukey’s multiple comparison test were used for statistical analyses (*p < 0.05).

Glycoprotein D from HSV-1 has been reported to localize at the nucleus of infected cells at structures described as replication factories (35) and to participate in primary and secondary envelopment of capsids from the nucleus to the Golgi apparatus (36). However, another study indicated that epithelial cells infected with an HSV-1 deletion mutant of gD released viral particles into the extracellular space and that these particles were phenotypically similar to those of the WT virus (23). To determine if DCs inoculated with ΔgD-2 released viral particles into the extracellular space and to evaluate their phenotype, we performed TEM of DCs inoculated with WT or ΔgD-2 virus, as well as their supernatants concentrated by ultracentrifugation. Overall, DCs inoculated with either virus displayed poor amounts of virus structures, which mainly consisted of non-enveloped capsids with and without an electron-dense content (Figure 3A). To determine if such viral particles were released into the extracellular media, we performed TEM (negative staining) of the ultracentrifugated supernatants recovered from DCs inoculated with the WT or ΔgD-2 virus. As shown in Figure 3B, we observed viral particles for both treatments (Figure 3B). To corroborate that these viral particles corresponded to newly synthesized viral structures, we also analyzed ultracentrifugated supernatants of cells treated with acyclovir and inoculated with these viruses. As expected, we were unable to recover sufficient viral particles to be observed by TEM (data not shown), suggesting that DCs inoculated with ΔgD-2 release newly produced virus particles into the extracellular space, regardless of the gD-2 deletion.

To gain further insight on the characteristics of the viral particles present in the ultracentrifugated supernatants of the DCs inoculated with the WT virus and ΔgD-2, as well as their amount relative to each other, we performed a western blot against the viral capsid protein ICP5 and a qPCR to quantify the number of viral genome copies in these structures. As shown in Figure 3C, virus-containing fractions recovered from the ultracentrifugated supernatants of DCs inoculated with ΔgD-2 contained more ICP5 than the fraction encompassing viral particles recovered from DCs inoculated with the WT virus (threefold difference). However, despite the increased levels of ICP5 detected in the virus fraction collected from DCs inoculated with ΔgD-2, as compared to DCs infected with WT virus, the number of viral genomes copies in these virus particles was inverted. As shown in Figure 3D, viral particles released by ΔgD-2-inoculated DCs contained significantly less viral genome copies than viral particles recovered from DCs inoculated with the WT virus, consistent with the virus phenotypes observed by TEM in DCs (Figure 3A). As expected, acyclovir treatment significantly reduced the amount of viral genome copies detected in the ultracentrifugated preparations, indicating that the virus particles recovered in these preparations were synthesized *de novo* by the inoculated DCs. Taken together, these results suggest that DCs inoculated with ΔgD-2 release defective viral particles into the supernatant and that these viral structures are mostly devoid of viral genome.

### WT but Not ΔgD-2 HSV-2 Induces Increased XBP-1 Splicing in DCs

**Figure 4:**
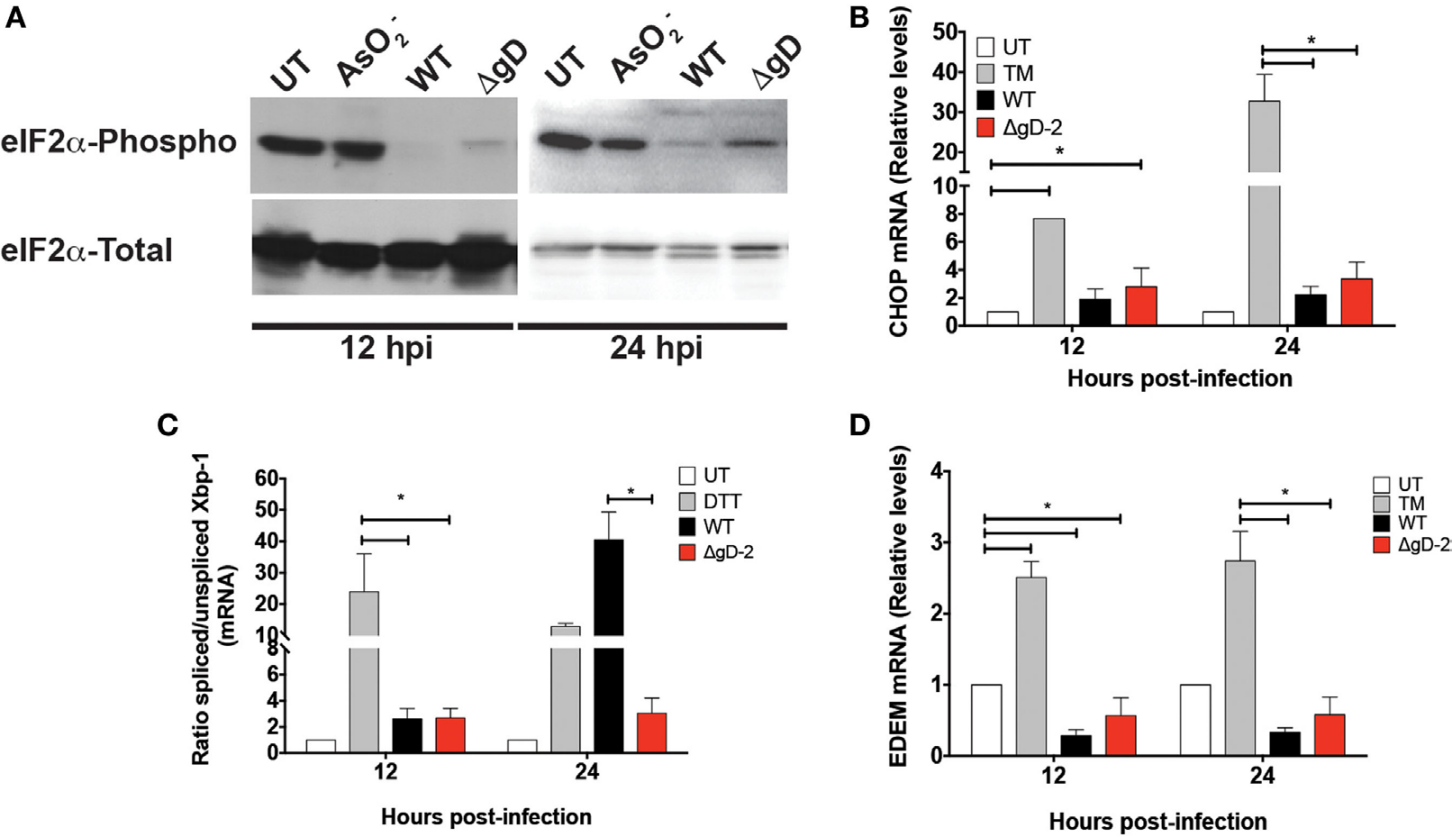
Differential Unfolded protein response in WT- and ΔgD-2 virus-inoculated dendritic cells (DCs). **(A)** Western blot analyses of the phosphorylation of eIF2-α at 12 and 24 h post-inoculation with the indicated treatments. ${\text{AsO}}_{2}^{-}$: sodium arsenite was used as a positive control for PERK activation and eIF2-α phosphorylation. **(B)** Relative expression of CHOP mRNA in virus-inoculated DCs at 12 and 24 h post-inoculation with the indicated viruses. Here, tunicamycin (TM) was used as positive control for ATF-6 activation. **(C)** Ratio of spliced to non-spliced XBP-1 mRNA in DCs at 12 and 24 h post-inoculation. Dithiothreitol (DTT) was used as a positive control for activating the IRE-1-α pathway. **(D)** Relative expression of EDEM in DCs at 12 and 24 h post-inoculation. The Western blots in **(A)** are representative of three independent experiments. Data for **(B)** and **(D)** are means ± SEM of five independent experiments. One-way analysis of variance and Tukey’s multiple comparison test were used for statistical analyses (*p < 0.05).

Because gD has been reported to be involved in viral egress replication cycle in infected cells and to localize at certain intracellular sites including the nucleus, perinuclear membrane, and rough endoplasmic reticulum (RER), we sought to assess whether the absence of gD in the mutant virus might trigger or modulate the UPRs in DCs (35, 36). Thus, we performed western blots and qPCR to analyze biomarkers associated with RER stress, namely those related to PERK, IRE-1, and ATF-6 signaling pathways. As shown in Figure 4A, both the WT and ΔgD-2 viruses blocked eIF2-α phosphorylation in DCs, indicating that the PERK pathway is modulated similarly by these viruses in these cells. Notably, the transcription of CHOP, which usually increase in response to PERK- and ATF-6-mediated UPR responses (37, 38), remained unaltered after infection with WT and ΔgD-2. However, a different result was observed for the splicing of XBP-1 mRNA, which is characteristic of the IRE-1 pathway (39). Whereas DCs inoculated with the WT virus displayed a significant increase in the splicing of XBP-1 mRNA at 24 h post-inoculation, this was not the case for ΔgD-2 (Figure 4C). Interestingly, this increase in XBP-1 mRNA splicing was not followed by an increase in EDEM mRNA transcription, which usually follows this pathway (Figure 4D) (40). Taken together, these data suggest that the WT and ΔgD-2 viruses elicit distinct UPR responses in DCs, which may account to some degree for the differences in viability and maturation processes described above.

### DCs Inoculated with the ΔgD-2 Activate CD8^+^ T Cells and CD4^+^ T Cells *In Vitro*

**Figure 5:**
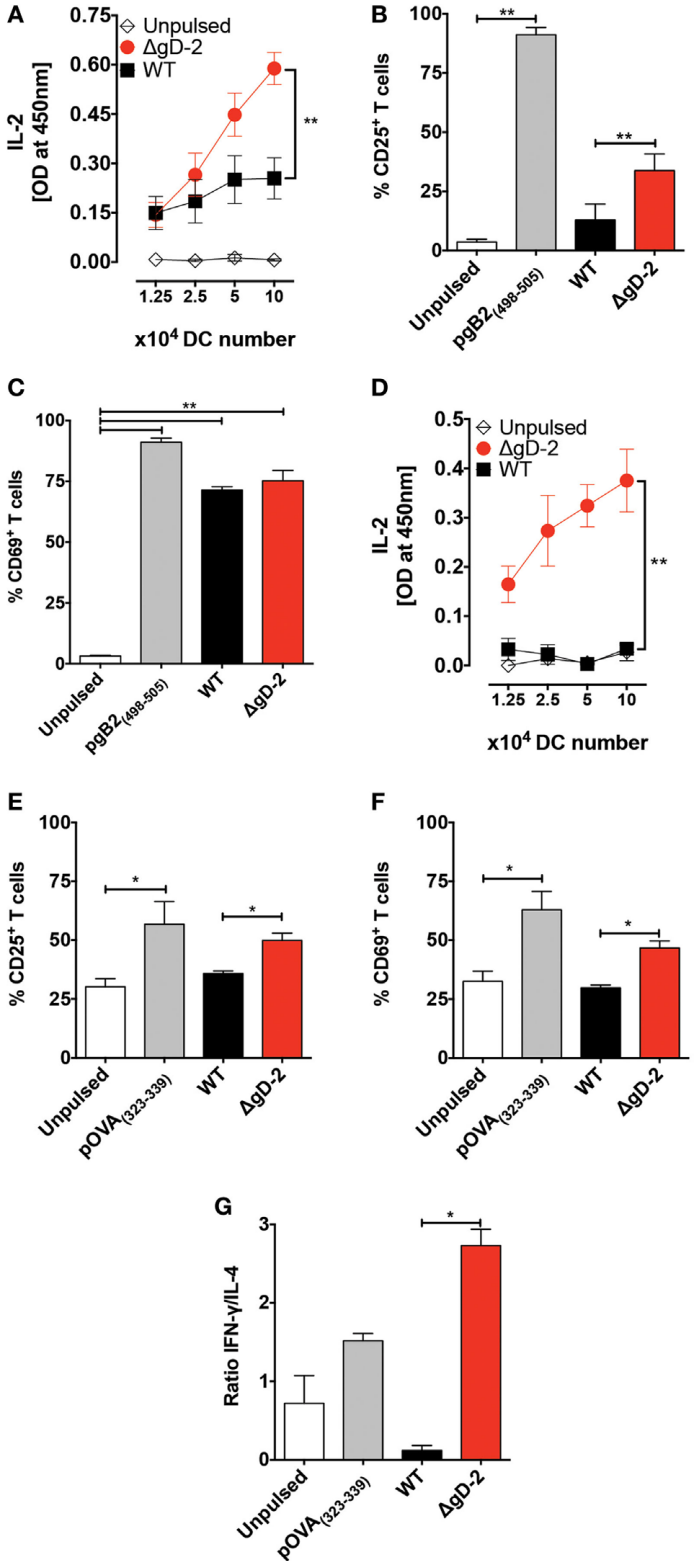
Dendritic cells (DCs) pulsed with ΔgD-2 activate CD4^+^ and CD8^+^ T cells *in vitro*. **(A)** IL-2 secretion in the supernatants of cocultures of virus-pulsed DCs with virus-specific gBT-I CD8^+^ T cells. **(B)** Surface expression of CD25 in T cells from cocultures of DCs and gBT-I cells. **(C)** Surface expression of CD69 in T cells from cocultures of DCs and gBT-I cells. **(D)** IL-2 secretion in the supernatants of cocultures of virus-pulsed DCs with OT-II CD4^+^ T cells. **(E)** Surface expression of CD25 in OT-II T cells from DC and T cell cocultures. **(F)** Surface expression of CD69 in OT-II T cells from DC and T cell cocultures. **(G)** Secretion of IFN-γ and IL-4 from OT-II CD4^+^ T cells in DC and T cell cocultures was measured by ELISA and is presented as a ratio. One-way analysis of variance and Tukey’s multiple comparison test were used for statistical analyses (*p < 0.05, **p < 0.01, ***p < 0.001).

To assess the capacity of DCs infected with ΔgD-2 to activate CD8^+^ and CD4^+^ T cells, we cocultured DCs with transgenic, antigen-specific T cells. As shown in Figure 5A, HSV-specific CD8^+^ gBT-I T cells, which recognize a peptide derived from gB, secreted significantly higher amounts of IL-2 when cocultured with DCs infected with ΔgD-2 compared to WT virus. We also observed significant increased CD25 expression by T cells that were cocultured with ΔgD-2-infected versus WT-infected DCs, consistent with T cell activation (Figure 5B). The expression of CD69, another marker of T cell activation, was upregulated in gBT-I T cells in all treatments where gB was present, consistent with a previous report (Figure 5C) (41).

To determine the capacity of DCs infected with ΔgD-2 to activate CD4^+^ T cells, infected DCs and T cells were cocultured using OT-II CD4^+^ T cells in the presence of their cognate ligand; as shown in Figure 5D, the cocultures with ΔgD-2-infected DCs secreted significantly higher amounts of IL-2 than did cocultures with WT-infected DCs. Similarly, T cells cocultured with ΔgD-2-infected DCs expressed higher levels of CD25 than did T cells cultured with WT virus-infected DCs (Figure 5E). A similar result was observed with CD69, indicative of T cell activation (Figure 5F). To determine the phenotype of these CD4^+^ T cells, we measured IL-4 and IFN-γ in the supernatants of the cocultures. Notably, CD4^+^ T cells cocultured with ΔgD-2infected DCs secreted more IFN-γ relative to IL-4 compared to T cells cocultured with WT HSV-2 infected DCs, consistent with a Th1-response (Figure 5G).

### ΔgD-2 Promotes Increased Migration of DCs into LNs and T Cell Activation *In Vivo*

**Figure 6:**
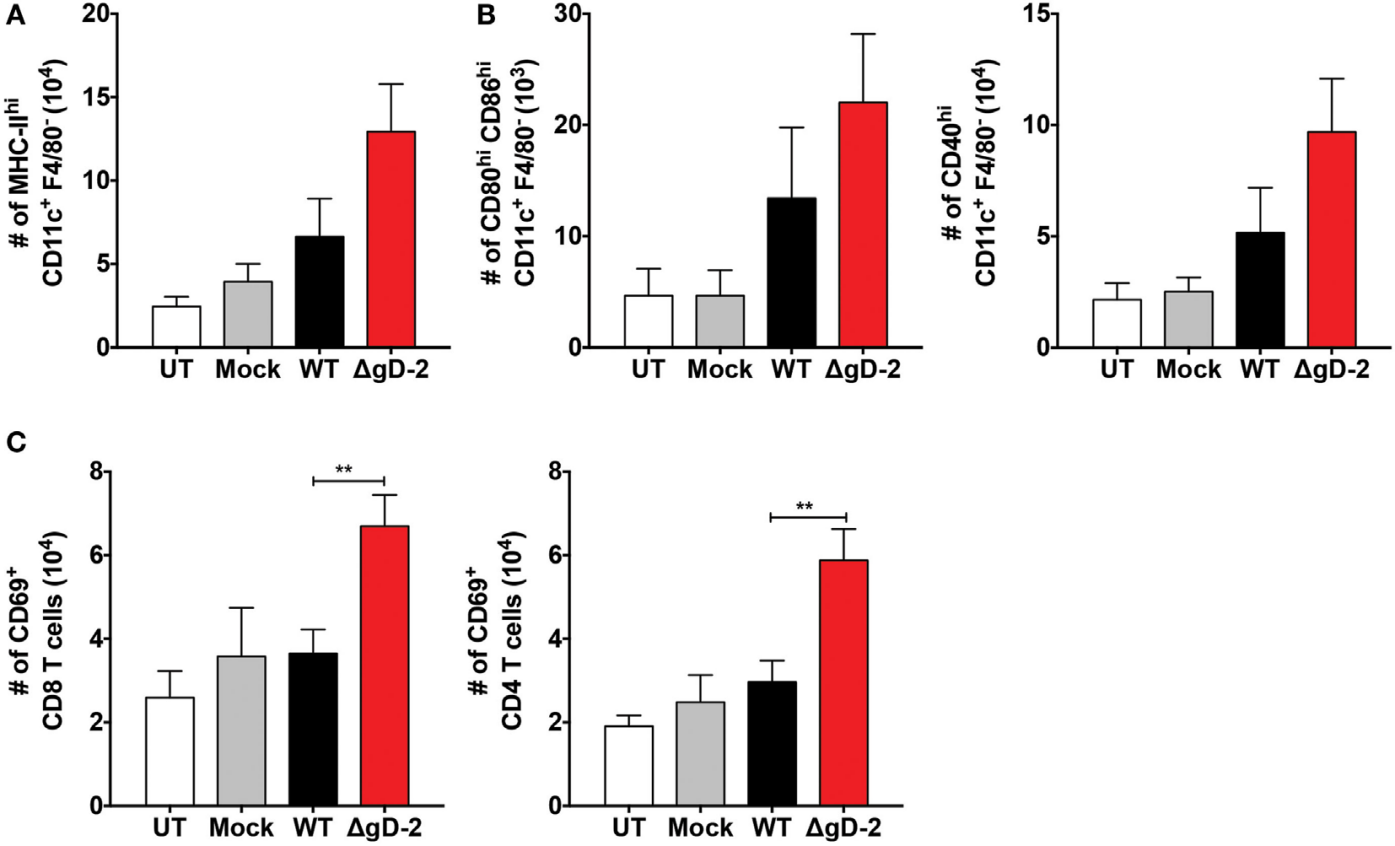
ΔgD-2 promotes dendritic cell (DC) migration and CD4^+^ and CD8^+^ T cell activation *in vivo*. **(A)** DC migration from the hind limb to inguinal lymph nodes (LNs) 48 h after subcutaneous injection of 10^6^ PFU of virus. **(B)** Phenotype of DCs in inguinal LNs after virus inoculation in the hind limb. The following surface markers were used: CD11c, F4/80, MHC-II, CD80, CD86, and CD40. **(C)** T cell numbers and phenotypes in inguinal LNs 48 after subcutaneous injection of 10^6^ PFU. One-way analysis of variance with Dunnett’s post-test was used. Data are means ± SEM of three independent experiments (*n* = 7–12 mice/group, **p < 0.01).

**Figure 7:**
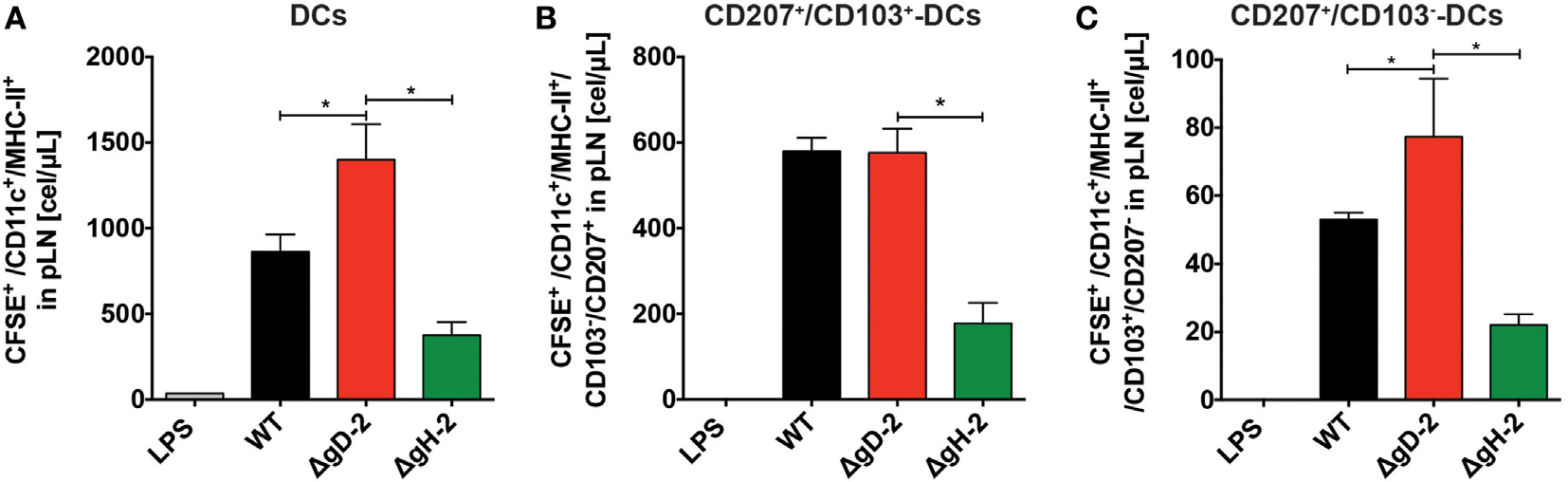
ΔgD-2 increases the relocation of CD103^+^ migrating dendritic cells (DCs) to draining lymph nodes (LNs). **(A)** Evaluation of DC migration from the footpads to popliteal LNs 24 h after injection of 10^6^ PFU of virus and CFSE tracking dye (CFSE^+^ gated, then CD11c^+^-MHC-II^+^ gated). **(B)** Quantification of CD207^+^-CD103^+^ migrating dermal DCs (CFSE^+^ gated, then CD11c^+^-MHC-II^+^ gated) relocating to the draining LNs after injection of 10^6^ PFU of virus and CFSE tracking dye. **(C)** Quantification of CD207^+^-CD103^−^ Langerhans cells (CFSE^+^ gated, then CD11c^+^-MHC-II^+^ gated) relocating to the draining LNs after injection of 10^6^ PFU of virus and CFSE tracking dye. One-way analysis of variance with Tukey’s post-test was used. Data are means ± SEM (*n* = 4 mice/group, *p < 0.05).

Because the activation of CD4^+^ and CD8^+^ T cells against HSV involves DC migration from the infection site to draining LNs (42), we assessed the migration of DCs *in vivo* after inoculation with ΔgD-2 and WT virus. As shown in Figure 6A, mice inoculated subcutaneously with ΔgD-2 near the hind flank displayed more DCs in the draining LNs than did animals infected with the WT virus 48 h after virus injection. Importantly, DCs recovered at this site after ΔgD-2 injection exhibited more enhanced maturation compared to DCs obtained from WT-inoculated mice, which was evidenced by greater surface expression of CD80^high^ -CD86^high^, and CD40 (Figure 6B left and right panel, respectively). Furthermore, 48 h after virus inoculation, animals infected with ΔgD-2 displayed higher numbers of activated CD8^+^ and CD4^+^ T cells in the draining LNs compared to mice infected with the WT virus (Figure 6C). Taken together, these data indicate that ΔgD-2 promotes DC activation and migration from the skin to LNs at higher levels than WT virus, which results in increased CD8^+^ T and CD4^+^ T cell activation *in vivo*. A similar result was obtained after inoculation of ΔgD-2 in the footpads of mice when DC were monitored in the draining popliteal LN using a tracking dye (CFSE) (33). As shown in Figure 7A, 24 h after virus injection, mice inoculated with ΔgD-2 displayed increased numbers of DCs in the corresponding LN when compared to mice infected with either WT or ΔgH-2 viruses. Analysis of the subtype of DC migrating to the LN revealed that ΔgD-2 significantly promoted the relocation of migrating CD103^+^ dermal DCs (dDCs), when compared to the WT virus (Figures 7B,C). ΔgH-2 overall elicited little DC migration.

### DCs Infected with ΔgD-2 Prime an Immune Response and Confer Protection against HSV-2 Challenge

**Figure 8:**
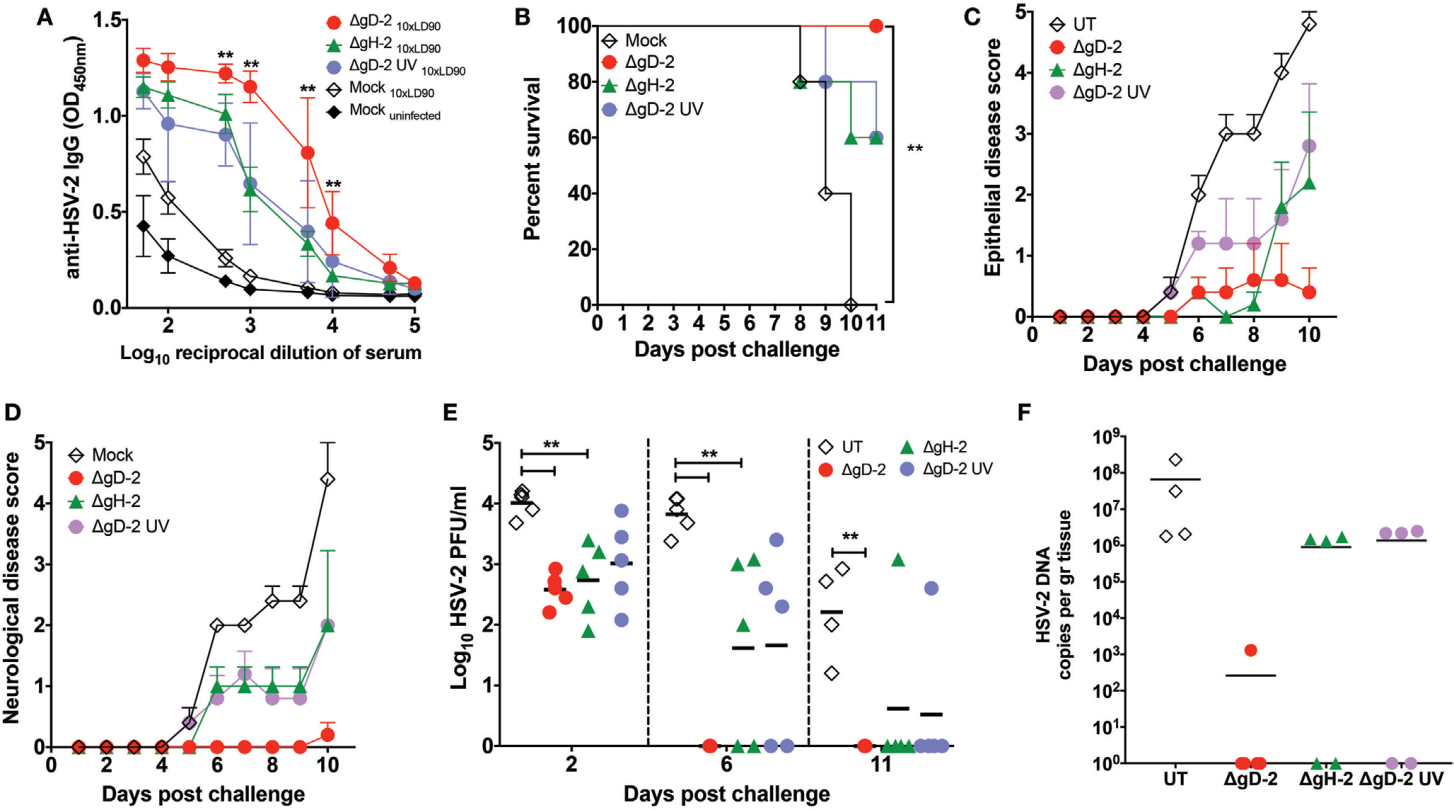
Transfer of dendritic cells (DCs) infected with ΔgD-2 confer protection against intravaginal lethal challenge with HSV type 2 (HSV-2). Mice were immunized subcutaneously with DCs infected *in vitro* with ΔgD-2, ΔgH-2, or UV-inactivated ΔgD-2 and administered three times over 7-day intervals and 2 weeks after the last dose challenged intravaginally with virulent HSV-2 (10× LD_90_). **(A)** Anti-HSV-2 antibodies in the serum of mice detected by ELISA at day 7 post-challenge. **(B)** Survival curves, **(C)** epithelial pathology score, 0: no disease; 1: slight erythema, edema; 2: moderate erythema, edema; 3: small lesion, hair loss; severe erythema, edema; 4: large lesion or multiple lesions, hair loss; severe erythema, edema, and **(D)** neurological pathology score, 0: no disease; 1: constipation; 2: hind limb paresis; 3: urinary retention; 4: hind limb paralysis; urinary retention; constipation after lethal challenge with HSV-2. **(E)** HSV-2 titers in vaginal lavages (VALs) at days 2, 6, and 11 (or at the time of euthanasia). **(F)** Quantification of viral genome copies in the dorsal root ganglia of mice injected with virus-treated DCs and challenged with a lethal dose of HSV-2 as determined by qPCR (*UL30* gene). Statistics was performed using two-way analysis of variance and Sidak’s post-test at 95% of confidence interval, data are means ± SEM (*n* = 5 mice/group **p < 0.05).

To determine whether DCs infected with virus *in vitro* can prime an immune response, we infected DCs *in vitro* with ΔgD-2, ΔgH-2, or UV-inactivated ΔgD-2 and transferred these cells thrice into mice before exposure to virulent HSV-2. Two weeks after the last injection of DCs, mice were challenged intravaginally with a 10-fold LD_90_ dose of virulent HSV-2 and followed for 11 days to determine disease progression and infection. As shown in Figure 8A, animals that received DCs inoculated with the abovementioned viruses displayed significant amounts of anti-HSV-2 antibodies, indicating that the transferred DCs were immunogenic. Importantly, mice that received ΔgD-2-infected DCs generated ~10-fold more antibodies against HSV-2 than animals that received DC treated with ΔgH-2 or UV-inactivated ΔgD-2, which may reflect the enhanced survival and/or trafficking to LNs of the ΔgD-2-infected DCs. As expected, animals that were not primed and received control DCs did not generate a significant antibody response. The antibody responses were reflected by the clinical response to viral challenge. Mice primed with virally infected DCs had reduced signs of epithelial and neurological disease and greater survival than controls, which resulted in a significant difference in survival comparing mice that received the ΔgD-2-infected DCs versus the control (Figures 8B–D). All (5/5) of the ΔgD-2-DC primed mice survived, whereas all of the mice that received the control DCs succumbed to the infection (Figures 8B–D). To follow infection in the genital tract, virus loads were determined in VALs at days 2, 6, and 11 (or at the time of euthanasia) by plaque assay on Vero cells. As shown in Figure 8E, all of the mice injected with ΔgD-2-infected DCs cleared the virus by day 6, compared to only 2/5 mice receiving ΔgH-2 or UV-ΔgD-2-infected and none of the control mice. Because HSV-2 infects neurons in the dorsal root ganglia to establish latency, we assessed viral loads in this tissue at day 11 post-challenge (or at the time of euthanasia) by qPCR. As shown in Figure 8F, mice primed with ΔgD-2-infected DCs manifested the lowest loads of virus in this tissue compared to the other groups. Finally, as expected animals that received untreated DCs manifested the highest viral loads in the genital tract and neurological tissue (Figures 8E,F). Taken together, these data suggest that DCs are capable of priming an immune response and the differential response of DCs to infection by ΔgD-2 compared to ΔgH-2 or WT virus may contribute to vaccine efficacy in this model.

## Discussion

HSV interferes with the function and viability of DCs, which may result in suboptimal antiviral responses allowing the virus to persist. Identifying how HSV interferes with DC function and promotes their death, may provide valuable insights for establishing protective immunity against these viruses (11, 15, 18). ΔgD-2 was restricted to a single cycle in DCs, similar to what has been described in other cell types, but in contrast to WT virus, did not induce cell death. Remarkably, ΔgH-2, which is also a single cycle virus behaved more like the WT virus and induced DC death. Noteworthy, a ΔgH-2 viral strain was not effective in a therapeutic vaccine clinical trial (43). Because WT virus propagated on VD60 cells (WT^+gD-1^) and ΔgD-2 propagated on ET60 cells (gD-2^−/+gD-2^) displayed similar phenotypes as their counterparts that express gD-2 and gD-1, respectively it is possible that newly synthesized gD-2, which is present in all the other viral strains may play a role in promoting DC death and dampening DC function. Overall the interaction between ΔgD-2 and DCs resulted in modest DC maturation, IL-6 was significantly increased after virus inoculation. IL-6 modulates the effector functions of numerous immune cells, either indirectly through the stimulation of acute phase responses (44) or directly by inducing, for example the differentiation of CD8^+^ T cells into cytotoxic T cells (45), promoting T follicular helper cell differentiation (46), or inhibiting TGF-β-induced Treg differentiation (47), among others. Interestingly, a previous study reported significant IL-6 secretion by cells that were inoculated with an HSV-2 virus that had mutations in the gD region that engages HVEM, suggesting that gD might interfere with intracellular signaling events that lead to the secretion of this cytokine (48). The absence of newly synthesized gD and the restriction to a single round of infection may allow DCs to sense the virus and mount an antiviral response capable of limiting its deleterious effects.

An important aspect of the ΔgD-2 virus is that its progeny does not express gD and thus are incapable of infecting other cells, such as bystander DCs in culture and T cells. This characteristic of this gD-mutant contrasts with WT HSV, which exerts inhibitory effects over T cells, by interfering with receptor signaling events and induces apoptosis (49–51). Additionally, gD *per se* (from HSV-1) has been reported to negatively modulate T cell proliferation, and thus, deleting this protein from the surface of DCs would likely alleviate some of the negative effects that gD has on T cells, if also applicable to gD-2 (gD from HSV-2) (50, 52). Consistently, CD45.1^+^ DCs cultured over ΔgD-2-inoculated DC were viable, unlike DCs cultured over similar cells infected with WT virus. These results also indicate that DC inoculation with ΔgD-2 does not impact the viability of bystander cells. Importantly, ΔgD-2- inoculated DCs activated CD8^+^ T cells and CD4^+^ T cells that displayed a Th1 phenotype, which contrasted with the Th2-like response observed for CD4^+^ T cells cocultured with WT virus-inoculated DCs. Previous reports propose that Th1 responses are likely optimal for HSV control in the mouse model and humans (53, 54), so it is noteworthy that a Th1-like antibody response was observed in ΔgD-2 vaccinated mice (6, 7).

Although gD from HSV-1 has been reported to localize at several cellular compartments and to play a role in virus egress (35, 36), our TEM images of virus-inoculated DCs overall revealed important similarities in the morphology of the defective viral particles released by WT- and ΔgD-2-virus-inoculated DCs and only differences in the frequency of a viral particle phenotype with alterations in the usually electron-dense core were observed. Importantly, qPCR quantification of viral genome content in the viral particles released by the ΔgD-2-inoculated DCs revealed that these structures were mostly devoid of viral genetic material, suggesting that gD may play a role in the assembly of viral particles released by immune cells. Noteworthy, it is likely that reduced viral genome content in the viral fractions collected from the ultracentrifugated supernatants of ΔgD-2-inoculated DCs was not due to reduced viral particle release by these DCs, as ICP5 a major HSV-2 capsid protein was abundantly detected in the viral particles recovered from these cells. However, we cannot rule out alterations in the amount of ICP5 expressed by the viral particles released by each of the treated DCs, which may account for the reported observations. These results and those from previous reports call for more detailed studies on the role and localization of gD-1 and gD-2 within immune and non-immune cells, as well as for studying the mechanism of exit of these non-enveloped particles from DCs.

Because the replication cycle of the ΔgD-2 virus may differ from that of the WT virus in modulating RER stress, we sought to evaluate UPRs in DCs inoculated with these viruses. As expected, the PERK response in DCs was similar for the WT and ΔgD-2 viruses, as both viruses express gB, which has been reported to be involved in modulating this pathway (55). However, an unexpected finding was the observation that, compared to ΔgD-2, the WT virus induced increased XBP-1 mRNA splicing in DCs. This result differs from that observed in a previous study with epithelial cells and HSV-1, in which no significant UPR response was observed after infection with the WT virus, suggesting that the UPR response differs depending on the cell type infected with HSV and possibly between HSV-1 and HSV-2 (56). Importantly, an increase in XBP-1 mRNA splicing has been previously associated with decreased cell viability in cells infected with the human cytomegalovirus (57), which overall decreases MHC-I presentation (58). Hence, the increase in XBP-1 mRNA splicing observed herein with the WT virus may contribute to increased DC mortality and reduced antigen presentation to CD8^+^ T cells. It will be relevant to assess whether this UPR pathway is involved in HSV-mediated DC death, together with other previously proposed mechanisms (18, 19). Moreover, interfering with the HSV-1 γ34.5 protein, which modulates the PERK UPR response by inhibiting eIF2-alpha phosphorylation and the beclin-1 autophagy pathway (59), was recently reported to produce an attenuated virus in DCs that conferred protective immunity against challenge (60). Thus, modulating the UPR response in DCs infected with HSV can promote cell survival and the induction of effective antiviral adaptive immunity.

Importantly, HSV infection interferes with the migration of Langerhans cells from the skin to LNs and induces their apoptosis (20). However, dDCs can capture apoptotic Langerhans cells and relay virus antigens to the LNs for activating CD4^+^ and CD8^+^ T cells (42, 61, 62). Here, we observed that subcutaneous injection of ΔgD-2 promoted DC migration to the draining LNs and that these migrating cells displayed a mature phenotype. Furthermore, ΔgD-2 inoculation promoted the relocation of migrating CD103^+^ dDCs to the LNs, which are known to be effective at cross-presenting antigens (42, 61, 62). Importantly, the ΔgH-2 virus did not promote significant relocation of DCs after subcutaneous inoculation. Furthermore, ΔgD-2 inoculation resulted in an increased early activation of CD4^+^ and CD8^+^ T cells, likely by CD103^+^ dDCs. Finally, priming of mice by immunizing with DCs infected with ΔgD-2 *in vitro* protected against a lethal intravaginal HSV-2 challenge resulting in reduced disease manifestations and viral loads in genital tract and in neurological tissues.

Taken together, our results suggest that the effectiveness of ΔgD-2 as a vaccine against HSV in the mouse model (6, 7) likely benefits, at least in part, from its ability to promote DC viability and migration to LNs, thus enabling effective CD8^+^ and CD4^+^ T cell activation *in vitro* and priming an immune response characterized by increased antibody responses and protection against disease *in vivo*.

## Ethics Statement

Mice were handled and euthanized according to the guidelines of the Comité de Bioética y Bioseguridad (Pontificia Universidad Católica de Chile) and according to the approved protocol CBB-201/2013.

## Author Contributions

AR-D, KW, PG, SB, and WJ designed experiments. AR-D, KW, ET, and MF conducted experiments. AR-D conducted experiments in Figures 1, 5, 7 and 8. KW conducted experiments in Figure 6. ET and MF provided help in conducting experiments in Figures 1 and 8. AR-D, PG, SB, KW, WJ, and BH analyzed the data and wrote the manuscript. All authors reviewed the manuscript.

## Conflict of Interest Statement

The authors declare that the research was conducted in the absence of any commercial or financial relationships that could be construed as a potential conflict of interest.
