# Supplementary material for: US6 Gene Deletion in Herpes Simplex Virus Type 2 Enhances Dendritic Cell Function and T Cell Activation
Source: Front Immunol. 2017 Nov 10;8:1523. doi: 10.3389/fimmu.2017.01523 (PMC5686121; doi:10.3389/fimmu.2017.01523)
Supplement: Supplementary file 1 [file table_1.docx]

Supplementary Material

*US6* GENE DELETION IN HERPES SIMPLEX VIRUS TYPE 2 ENHANCES DENDRITIC CELL FUNCTION AND T CELL ACTIVATION

Angello Retamal-Díaz^1^, Kayla Weiss^3^, Eduardo Tognarelli^1^, Mariela Freire^1^, Susan M. Bueno^1,2^, Betsy C. Herold^3,4^, William R. Jacobs Jr.^3,5,6,7^, and Pablo A. González^1,*^.

*** Correspondence:**Dr. Pablo González, Millennium Institute on Immunology and Immunotherapy, Departamento de Genética Molecular y Microbiología, Facultad de Ciencias Biológicas, Pontificia Universidad Católica de Chile, Santiago-Chile. Av. Portugal 49, Santiago E-8330025, Chile. E-mail: pagonzalez@bio.puc.cl

# 1. Supplementary Tables

**1.1 Supplementary Table 1**

**Table S1. Primers used to generate the ΔgI and ΔgJ deletant viruses.**

| **Region amplified** | **Primer Name** | |  | **Sequence (5' to 3')** | |
| --- | --- | --- | --- | --- | --- |
| **a. Primers for constructing p0004S-**Δ**US5 and p0004S-**Δ**US7 (*Van91I sites underlined)*** | | | | | |
| *Region homologous to the left of US5* | LL-Van91I-US5 | |  | TTTTTTTT CCATAAATTGG CCT CCG CCG CGC CCA C | |
|  | LR-Van91I-US5 | |  | TTTTTTTT CCATTTCTTGG TGA GAC CAA AAC AAC AGG CAC GCC CC | |
| *Region homologous to the right of US5* | RL-Van91I-US5 | |  | TTTTTTTT CCATAGATTGGTTT TTC GCC TTT CTG GCC TTG CCC CC | |
|  | RR-Van91I-US5 | |  | TTTTTTTT CCATCTTTTGG CGA TGG GGC ATG TAG GAG CAC GCT G | |
| *Region homologous to the left of US7* | LL-Van91I-US7 | |  | TTTTTTTT CCATAAATTGG TAT CGC ATG GGA GAC AAT TGC GCT ATC CCC A | |
|  | LR-Van91I-US7 | |  | TTTTTTTT CCATTTCTTGG ACC GGG ATC GGA CGG GTC GAG | |
| *Region homologous to the right of US7* | LR-Van91I-US7 | |  | TTTTTTTT CCATAGATTGG GTC CAA GCG GGC CGT TCG GCA G | |
|  | LR-Van91I-US7 | |  | TTTTTTTT CCATCTTTTGG CGT CAC GCC CGC GTC GTC TTC TTC | |
| **b. Primers for verifying deletion of gI and gJ** | | | | | |
| *Left to US5* | gJ2-L-check | |  | CGG GCC TCC GAC CCG GTA CG | |
|  | *sacB-*out | |  | AGG ATA CAG GAC CTG CCA AT | |
|  | |  | | |  |
| *Right to US5* | gJ2-R-check  *hyg-*out | |  | CAG CTC GAT GAT GCC GTC GTA GTA GTT TGT ATG G  CTT CAC CGA TCC GGA GGA AC | |
| *Left to US7* | gI2-L-check | |  | CAA CAA ACG CGA CCG CAC GCC TC | |
|  | *sacB-*out | |  | AGG ATA CAG GAC CTG CCA AT | |
| *Right to US7* | gI2-R-check  *hyg-*out | |  | GGC AGG GTC TCT GGC GGG TCC  CTT CAC CGA TCC GGA GGA AC | |
|  |  | |  |  | |
