# Supplementary figures and images for: US6 Gene Deletion in Herpes Simplex Virus Type 2 Enhances Dendritic Cell Function and T Cell Activation
Source: Front Immunol. 2017 Nov 10;8:1523. doi: 10.3389/fimmu.2017.01523 (PMC5686121; doi:10.3389/fimmu.2017.01523)

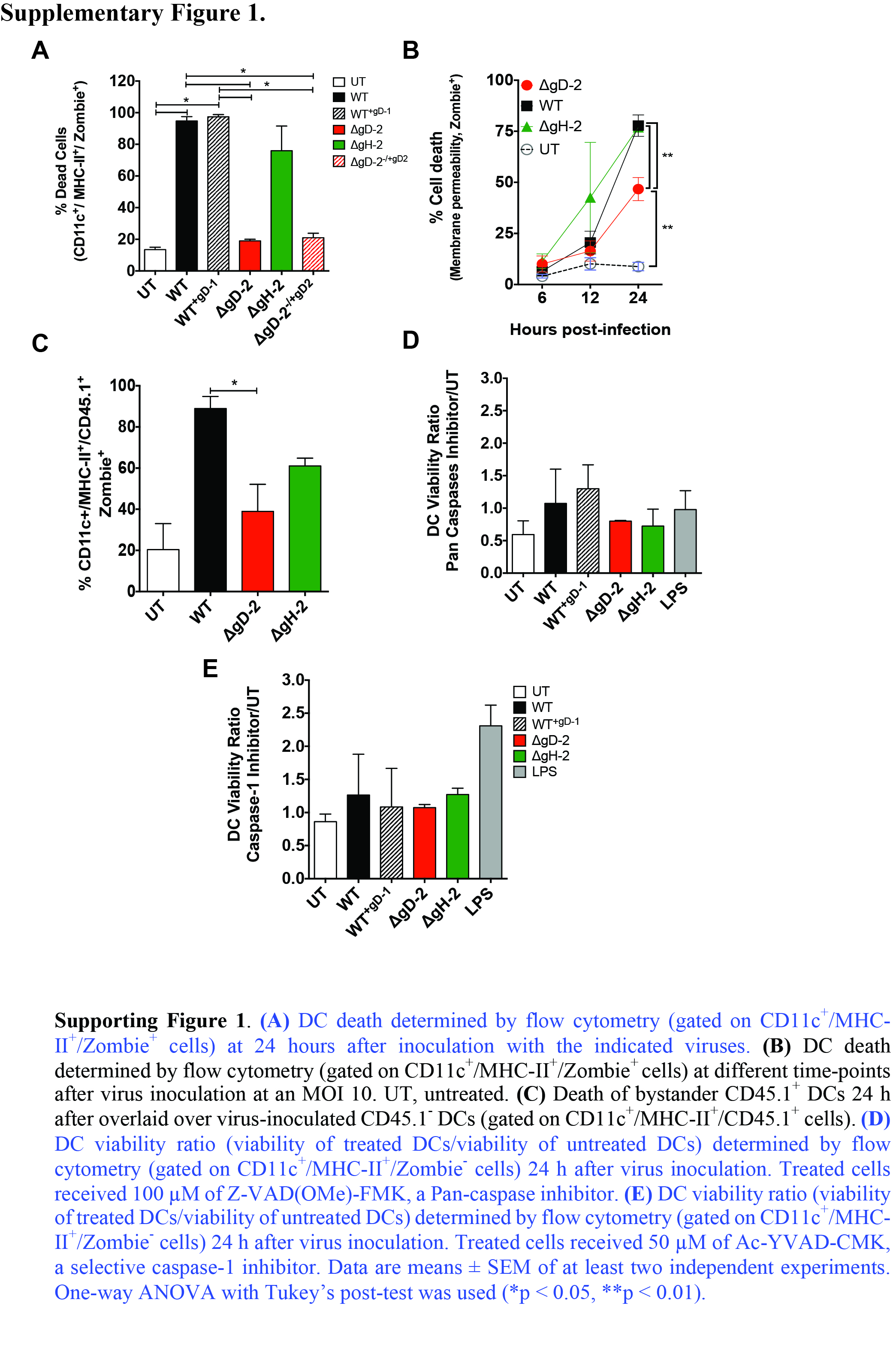

Supplement: Supplementary file 2 [file image_1.jpeg]

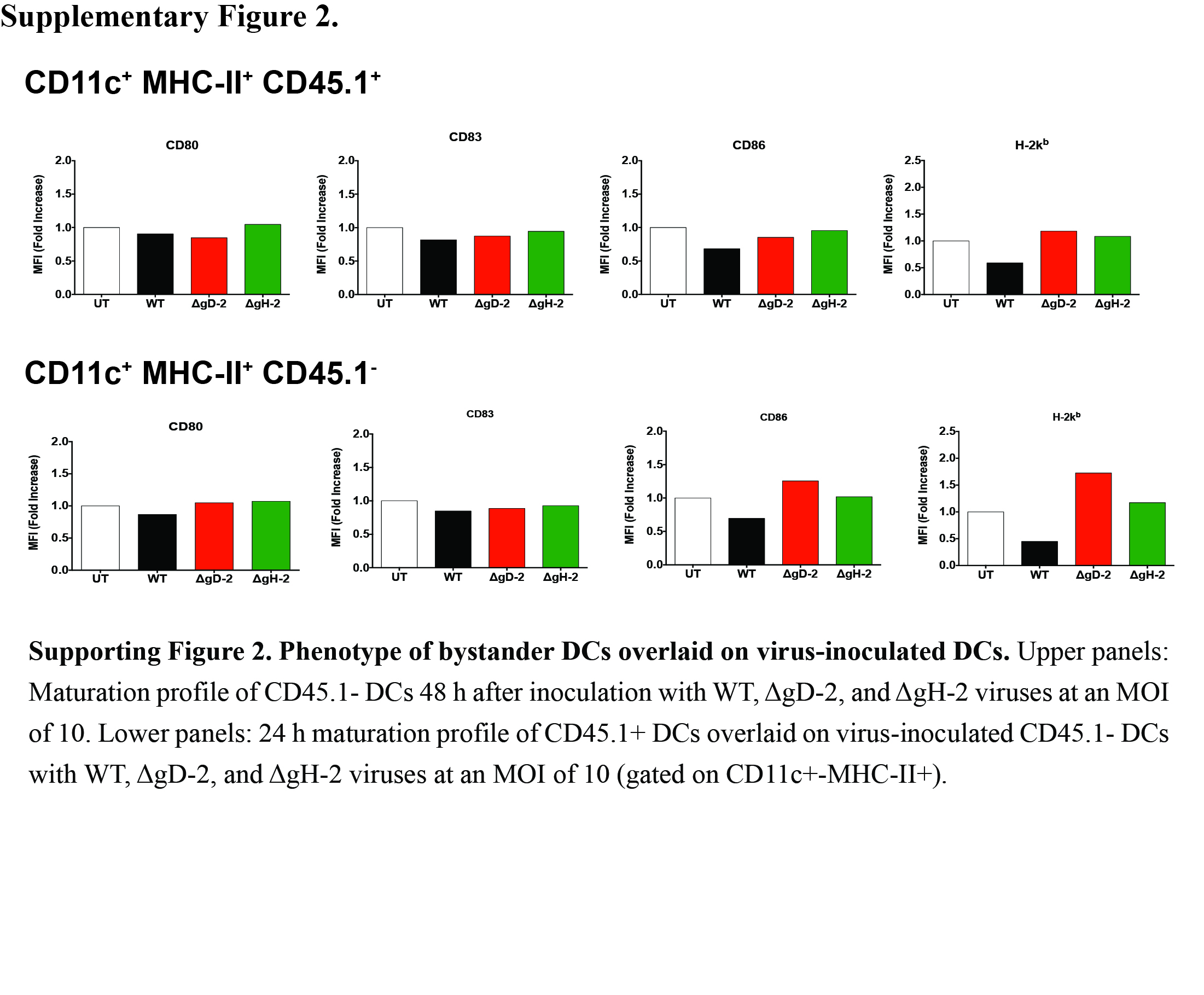

Supplement: Supplementary file 3 [file image_2.jpeg]

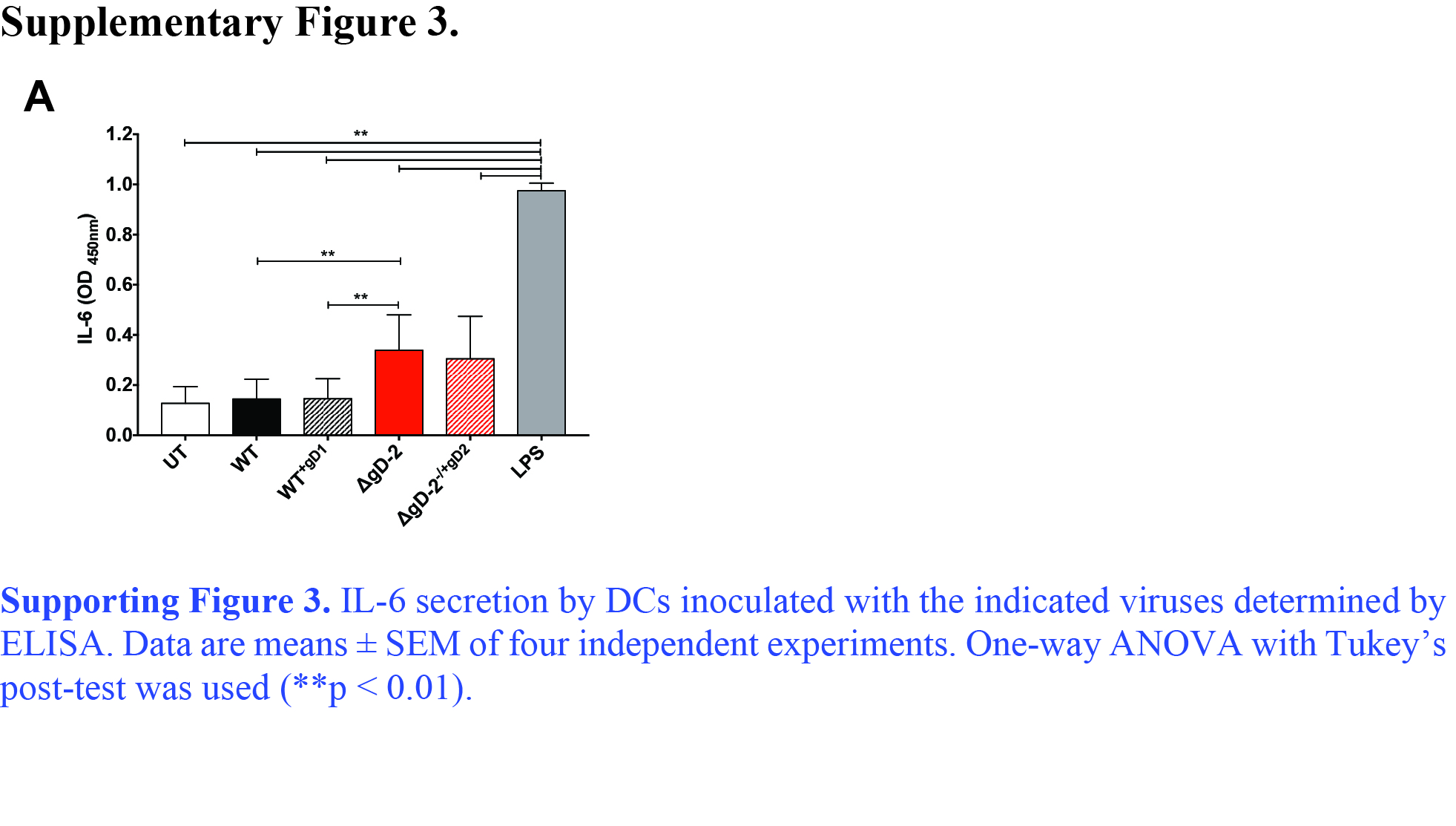

Supplement: Supplementary file 4 [file image_3.jpeg]
